# Supplementary material for: P-TRAP: a Panicle Trait Phenotyping tool
Source: BMC Plant Biol. 2013 Aug 29;13:122. doi: 10.1186/1471-2229-13-122 (PMC3848748; doi:10.1186/1471-2229-13-122)
Supplement: Additional file 7 — P-TRAP source code. The Netbeans project that contains the source code of the application. [file 1471-2229-13-122-S7.zip › souce.zip!/P-TRAP source code/Grain/doc/javadoc/org-bioalg-configurationfilesprocessor/index-all.html]

Index (ConfigurationFilesProcessor)


JavaScript is disabled on your browser.


- Package
- Class
- Tree
- Deprecated
- Index
- Help

*org.bioalg.configurationfilesprocessor 1.0*

- Prev
- Next

- Frames
- No Frames

- All Classes

G O X 


## G

getIntValue(Element, String) - Static method in class org.bioalg.configurationfilesprocessor.XMLConfigurationParser


getTextValue(Element, String) - Static method in class org.bioalg.configurationfilesprocessor.XMLConfigurationParser


getXMLDocument(File) - Static method in class org.bioalg.configurationfilesprocessor.XMLConfigurationParser


getXMLDocument(InputStream) - Static method in class org.bioalg.configurationfilesprocessor.XMLConfigurationParser


## O

org.bioalg.configurationfilesprocessor - package org.bioalg.configurationfilesprocessor


## X

XMLConfigurationParser - Class in org.bioalg.configurationfilesprocessor


XMLConfigurationParser() - Constructor for class org.bioalg.configurationfilesprocessor.XMLConfigurationParser

G O X

- Package
- Class
- Tree
- Deprecated
- Index
- Help

*org.bioalg.configurationfilesprocessor 1.0*

- Prev
- Next

- Frames
- No Frames

- All Classes
